# Books and Authors

**Published:** 1912-03

**Authors:** 


### BOOKS AND AUTHORS

### Recent Progress in Orthopedic Surgery.

LEONARD ELY, in his recent work on Joint Tuberculosis, (see Review, September, 1911), gives some positive views of his own, developed from special study and research. Tuberculosis of a joint exists in the red marrow and synovia only; after true ankylosis occurs, these tissues disappear by degrees, the bone changes in structure and sometimes a central canal forms. The best cures of tuberculosis have been seen after resections which have provided complete fixation. Hence he believes that incomplete fixation, as provided by braces, etc., is not nearly so effective in curing the process as complete fixation. The best illustration is in the knee of the adult, where he does a modified resection, taking off very little bone and cutting very few ligaments. Bony ankylosis is secured with the loss of very little tissue. At the hip through an anterior incision, he chisels out the head of the femur, and puts up the leg in abducton to prevent dislocation, expecting an early cure.

In Pott's disease, all methods, excepting perhaps Calot's, have been inefficient, from the standpoint of preventing deformity. A kyphos will form and enlarge, especially in the dorsal region, in spite of supports. The first attempt to prevent deformity by operation was by wiring the spinous processes together. Next Lange introduced metal splints alongside the spinous processes, securing them in place by silk. Hibbs operated on several cases by cutting the spinous processes loose at the base, tilting them downward so that each reached from the base above to touch the base below by its tip; the object being to cause a stiffening at that site and prevent the formation of a kyphos. Albee split the spinous processes and inserted into the interval a piece of bone from the tibia, and he reports three cases. Whitman, Annals of Surgery, believing the indication is for the dorsal region chiefly, operated on one case, removing a long stout piece of bone from the tibia, and inserting it alongside the spinous processes, which were freshened to cause bony union. These cases are all supposed to go unsupported after a short time, the bony union causing an ankylosis, and a local cure.

On account of the universally poor results in structural lateral curvature by the usual methods, more articles constantly appear, in which by various means, forcible correction of the deformity is being attempted. Plaster jackets, applied in various ways, in varied positions, are being used. The keynote of success will probably be that the case must lie flat on the back until the jacket with the use of force has corrected or overcorrected the twist; then the upright position with the effect of gravity allowed.

In infantile paralysis the use of silk ligaments is gaining greatly in favor. The idea is this: wherever there is an unstable joint, insert if possible, strands of braided silk, from bone to bone. Later, on functional use, these strands become imbeded in fibrous tissue, which strengthen as stress is thrown on them. Thus new ligaments are formed which render the joint more stable and prevent deformity. The most usual site for this operation, is at the ankle, where silk is introduced to prevent toe drop.

For the spastic paraplegia of Little's disease, instead of tenotomies of the contracted tendons, or resections of the posterior nerve roots, Allison and others are paralyzing the nerves governing the spastic muscles by alcohol injections. For example, in a case with crossed thighs, flexed knees, and pointed feet, the nerves sought for and injected would be the objurators, the sciatic branches to the hamstrings, the branches of the internal popliteal to the calf muscles. The spasticity is at once lost, the opposing muscles act and gain in strength, and the case soon walks, without spasticity. The operations are of greater magnitude than tenotomies but the results seem to justify this.

---

**Practical Electro-Therapeutics and X-Ray Therapy, Dr. J. M. Martin,** Dallas, Tex., published by the C. V. Mosby Co., St. Louis, 1912, 446 pages, 219 illustrations, $4.00.

THE Mosby Co. have a reputation for finding authors who prepare works of practical value and bringing the knowledge on a subject up to date, in convenient form. They are no respectors of persons or places, but appeal to physicians who will form their own opinions and who demand as authority, the test tube, laboratory, and clinic, rather than some one's ipse dixit. Dr. Martin presents his subject in a plain, forcible way, with more than enough scientific foundation for the practical applications of electricity and X-rays but paying special attention to the daily needs of the practitioner.

**Medical Jurisprudence and Toxicology,** by the late **Dr. John J. Reese,** 8th edition revised by **Dr. D. J. McCarthy of Philadelphia,** published by P. Blakiston's Son & Co., Philadelphia, 1911. 660 pages, $3.

IDENTIFICATION of dead bodies, determination of cause and time of death, sexual problems, insanity, life insurance, etc., are discussed in orderly sequence nearly half of the work being devoted to toxicology. It is a concise, thorough treatise.

---

**Infections of the Hand, Dr. Allen B. Kanavel,** Chicago, published by Lea & Febiger, N. Y. and Philadelphia, 1912. 447 pages, 133 engravings. $3.00.

PROBABLY most of our readers, at first thought, would express the opinion that so detailed and local a surgical problem does not warrant a text book of this size. On analysis, this opinion means that the average physician does not realize the complexity and importance of the subject. The book itself demonstrates its right to exist and we feel sure that its use will result in fewer permanent cripples and fewer deaths.

---

**The Champlain Tercentenary,** Report prepared by **Hon. Henry Wayland Hill,** LL.D., Secretary of the Commission. Printed at Albany, 1911, by the J. B. Lyon Co., State Printers. 534 pages, illustrated.

THE value of such reports to future historians can scarcely be overestimated and, very properly, this report contains not only the history of three centuries ago but much interesting general information, as well as the history of the tercentenary celebration itself. Our distinguished fellow townsman, Senator Hill, has fulfilled most ably, the difficult task laid upon him. Such a task requires literary ability, much critical knowledge both of history in the narrow sense of a record of events, and in the broader sense of a study of human forces

---

**History of the Movement for the State Registration of Nurses. Dr. Eugene Underhill** of Philadelphia; Reprint from the Journal of the Medical Society of New Jersey, 1911. 24 pages, paper, free.

This review of a recent movement in various states, deserves careful consideration. If the unfavorable view of the author is correct, we need to unmake some laws; if incorrect, a great injustice is done to a sister profession or, at least, to an important element in it. The pamphlet is, however, by no means an attack on nurses in general. On the contrary it takes up the cudgel in their defense against . . . "the petty, nagging, inhuman, fiendish grind of one set of women upon another," in hospitals, demands greater regard for the health and strength of pupil nurses and elimination of menial tasks from their routine. We also approve of the view that a state board examination, such as is quoted from the records of New York, asking for treatment, of one disease after another, tends to develop a meddlesome nurse—of the type of popular magazine stories, for instance. We would advise those specially interested to send for the pamphlet and give it their attention.

---

**Handbook of Physiology, W. D. Halliburton**, M.D., LL.D., F.R.C.P., F.R.S., London, published by P. Blakiston's Son & Co.. Philadelphia, 1911, 10th edition, 923 pages, 592 illustrations, $3.00.

The work begins with a brief but quite thorough discussion of histology and cellular physiology and ends with a similar discussion of embryology. It is systematically arranged and, without attempting to enter into minutiae, is a full and up-to-date treatise.

---

**Twenty-Seventh Annual Report of the Bureau of American Ethnology** for the year 1905-6, printed at the Government Printing Office, Washington, 1911. 672 folio pages, illustrated. This volume, prepared under the direction of **W. H. Holmes**, Chief of the Bureau, consists mainly of an elaborate monograph on the customs, music and habits of the Omahas, by **Alice L. Fletcher** and **Francis La Flesche,** the latter being a member of the tribe.

The value to posterity, not to mention the living, of such thorough studies of the American aborigines, is incalculable, especially in view of the fact that but little time remains in which such studies will be possible and that, with possibly a few exceptions, there are no longer to be found, illustrations of primitive man, of the stage of savagery and living in the stone age. The relatively small amount of money required for scientific research warrants the development of a public sentiment in favor of some slight retrenchment in military and other expenditures, and the appropriation of sufficient funds to publish the reports of this institution up to date.

We have known personally, especially during the Pan American exposition, the devotion and scholarship of Prof. Holmes and of Miss Fletcher and of many of their colleagues.

While this particular volume does not appeal, except in some details, especially to the medical profession, it is worth reading as a matter of general educational interest. We take this occasion to repeat what has so often been said, that every professional and business man .should have some hobby aside from his work and we suggest, particularly to country physicians that their knowledge of anatomy, habits of study and local acquaintance, render archaeology of the American aborigines a most appropriate and fascinating form of diversion. With very little effort and loss of time, they can do a great deal toward preserving information concerning the former occupants of this country.

**Retinoscopy, James Thorington,** A.M., M.D., Philadelphia, published by P. Blakiston's Son & Co., Philadelphia, 1911. 6th edition, 70 pages, 61 illustrations, 10 colored, $1.00.

This is a very concise, practical treatise, of interest both to the general practitioner and the ophthalmologist.

---

**Manual of the International List of Causes of Death.** Prepared by the Bureau of the Census.

While a great deal remains to be done in systematic nomenclature of disease, this list tabulates in convenient form the present accepted nomenclature and should be consulted by all physicians, especially in regard to death certificates, in which accuracy and uniformity are important. We understand that the Manual is intended for free distribution.

---

**The Practical Medicine Series.** Ten Volumes. Under the general editorial charge of **Gustavus P. Head,** M.D. Vol. X, Nervous and Mental Diseases. Edited by **Drs. Hugh T. Patrick** and **Peter Bassoe.** Series 1911. Chicago: The Year Book Publishers. (Price, $1.25 each; entire series, $10.00.)

An excellent and practical review of recent current medical literature, carefully classified for convenience in "reading up."

---

**Practice of Medicine, Sir Wm. Osler,** M.D. Published by D. Appleton & Co., 7th edition, 1,143 pages, illustrated. $ .

The title, the author's name and the fact that this is the seventh edition, render comment superfluous. Osler's Practice is a standard work, both for students and practitioners, it has found favor in the past both on account of the authority with which it is entitled to speak and on account of its practical excellence. To add our recommendation, would be like endorsing Rockefeller's check.

---

**Static Joint Diseases. Dr. George Preiser,** Hamburg. A monograph, 278 pages, 272 illustrations. 1911. Verlag von Ferdinand Enke Stuttgart, Germany.

The author makes a very good case for a reasonable classification and treatment of chronic non-inflammatory localized joint conditions with and without bony changes, often termed "local arthritis deformans."

He groups normal joints according to their relations with neighboring joints into static (mechano- functinal) unities in which a static disturbance of one joint in the unity makes itself felt more or less in all the rest. The most important feature of such disturbances, is an alteration of the normal opposition of cartilaginous joint surfaces, throwing certain surfaces or parts of them, permanently out of function leaving the rest of the joint

